# Mesenchymal stem cell mediates cardiac repair through autocrine, paracrine and endocrine axes

**DOI:** 10.1186/s12967-020-02504-8

**Published:** 2020-09-01

**Authors:** Celia Sid-Otmane, Louis P. Perrault, Hung Q. Ly

**Affiliations:** 1 Department of Pharmacology and Physiology, Université de Montréal, Montreal, QC Canada; 2 Department of Medicine, Université de Montréal, Montreal, QC Canada; 3 Research Centre, Montreal Heart Institute, Université de Montréal, 5000 Belanger Street, Montreal, QC H1T 1C8 Canada; 4 Department of Cardiovascular Surgery, Montreal Heart Institute and Université de Montréal, Montreal, QC Canada

**Keywords:** Paracrine, Autocrine, Endocrine, Mesenchymal stem cells, Remote delivery

## Abstract

In the past decade, despite key advances in therapeutic strategies following myocardial infarction, none can directly address the loss of cardiomyocytes following ischemic injury. Cardiac cell-based therapy is at the cornerstone of regenerative medicine that has shown potential for tissue repair. Mesenchymal stem cells (MSC) represent a strong candidate to heal the infarcted myocardium. While differentiation potential has been described as a possible avenue for MSC-based repair, their secreted mediators are responsible for the majority of the ascribed prohealing effects. MSC can either promote their own survival and proliferation through autocrine effect or secrete trophic factors that will act on adjacent cells through a paracrine effect. Prior studies have also documented beneficial effects even when MSCs were remotely delivered, much akin to an endocrine mechanism. This review aims to distinguish the paracrine activity of MSCs from an endocrine-like effect, where remotely transplanted cells can promote healing of the injured myocardium.

## Background

Ischemic heart disease (IHD) due to coronary artery disease remains a serious burden on health systems across Western countries. Medical advances and device-based therapies have impacted mortality and improved quality of life of such patients [1]. These therapies are designed to rescue the ischemic but viable tissue only and fail to address the key molecular targets participating in the pathological cardiac remodeling [2]. Cardiomyocytes being terminally differentiated with minimal regenerative ability (0.5–2%), cardiac transplantation remains the only true cure for failing hearts [3]. However, the limited number of available donors limits the impact of such a therapeutic avenue. Tissue regeneration has emerged as a promising field of research using mainly cell-based therapy [4].

Embryonic and adult stem cells are capable of generating new tissue through differentiation into multiple lineages. Embryonic stem cells (ESC) isolated from the inner cell mass of blastocytes are pluripotent and capable of generating the three germinal layers [5]. However ethical issues and teratoma formation limit their clinical use. Induced pluripotent stem cells (iPSC) have very similar characteristics to ESC where terminally differentiated cells have been used to generate pluripotent cells [6]. Clinical translation of iPSC overcomes ethical issues related to ESC but security concerns with teratoma formation hinder their clinical use [7].

Of the adult stem cells, MSCs represent an interesting population that garnered increased interest for clinical translation in the past decade. First identified and isolated from bone marrow, stromal stem cells have now been isolated from various organs such as placenta, cord blood or adipose tissue [8]. When isolated from adipose tissue, they are called adipose derived stem/stromal cells (ASCs) and have become attractive candidates for cell-based therapy as they are readily and more easily isolated while engendering minor donor discomfort, compared to their bone marrow derived counterparts. Furthermore, adipose tissue offers higher quantities of stem cells compared to bone marrow [9]. At first, the self-renewal and differentiation of stromal stem cells were the main reasons for their use in regenerative medicine. Moreover, their low immunogenicity and documented immunomodulatory properties [10] prompted the possibility to have a bank of cells available for allogenic transplantation for “off the shelf” use in various clinical conditions. The major limitation in their therapeutic efficacy however has been their low engraftment after transplantation [11]. Nevertheless, in the last decade, conflicting results on engraftment percentage prompted a debate as to whether engraftment was mandatory to the therapeutic efficiency. It is very unlikely that the low engrafted rate of cells explains the reported therapeutic impact in both preclinical and clinical studies [12]. The trophic and immunomodulatory properties of MSCs are now believed to be the main mechanism underpinning the therapeutic impact in preclinical studies [13]. Unfortunately, there remains discrepancies between animal models and clinical studies that hamper the transition from bench to bedside. Unraveling key modulators in the secretome will promote successful clinical transition.

**Fig. 1:**
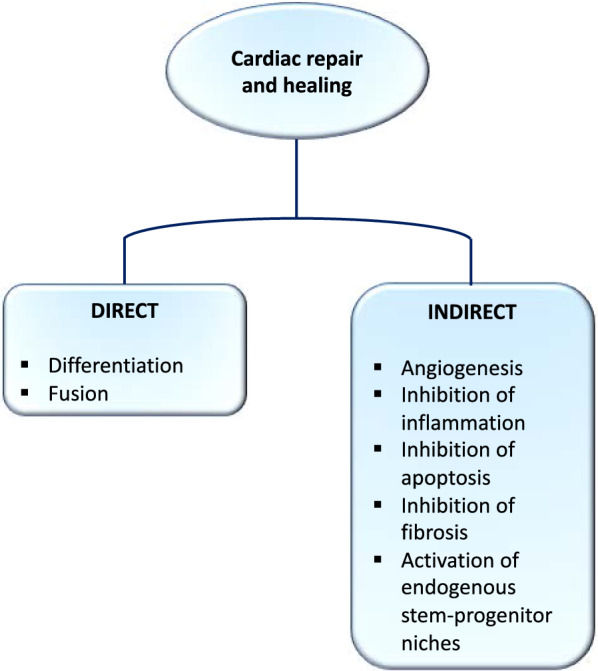
Cardiac healing and repair mediated by direct and indirect mechanisms in cardiac cell therapy

In order to heal after an ischemic episode, different processes need to act in concert (Fig. 1). Cardiac cell-based therapy can either act directly through transdifferentiation and fusion to replenish the lost tissue or indirectly by promoting angiogenesis, immunoregulation and inhibiting apoptosis and fibrosis through released factors [14]. Various clinical trials have been conducted using MSCs or ASCs in cardiovascular disease. Studies have examined both autologous and allogeneic cell transplantation (summarized in Table 1). The POSEIDON study concluded on comparable safety and efficacy between allogeneic and autologous MSCs [15]. The PRECISE study was the first randomized placebo-controlled trial showing feasibility and safety of transendocardial administration of ASCs [16]. Adverse effects have been rarely reported and cells showed some efficacy in improving cardiac function. However, better knowledge of parameters such as delivery route, cell dosage and appropriate timing for administration can substantially improve effectiveness [17]. Rushing translation to clinical application despite poor understanding of the biological mechanisms have yielded heterogenous efficacy outcomes. Investigating the kinetics of cell or derived components delivery is still a challenge. There is a lack in defining the best scenario between early and late administration, balancing between risk of toxic microenvironment for the injected material early-on after reperfusion or massive tissue damage because of delayed administration. From clinical studies, it was demonstrated that the most efficient time window for treating myocardial infarction is within a week after reperfusion [18]. More trials should follow this lead in order to confirm this time window.

**Table 1:** Clinical benefits from key clinical trials using stromal stem cells in heart diseases

Clinical trial	Year	Cell type	Patient population	Delivery route	LVEF	LVEDV	Infarct size
POSEIDON [15]	2012	Allogeneic and autologous BM-MSC	ICM LVEF ≤ 50%	Transendocardial	↑	↓	↓
APOLLO [66]	2012	Autologous ASC	STEMI	Intracoronary	↔	N/A	↓
C-CURE [67]	2013	Autologous BM-MSC	LVEF 15–40%	Endoventricular	↑	↓	N/A
PROMETHEUS [68]	2014	Autologous BM-MSC	ICM	Intramyocardial	↑	↓	↓
PRECISE [16]	2014	Autologous ASC	ICM, CABG	Transendocardial	↔	↔	↔
Gao et al. [69]	2015	Allogeneic WJ-MSC	STEMI	Intracoronary	↑	↓	N/A
TRIDENT [70]	2017	Allogeneic BM-MSC	ICM, LVEF ≤ 50%	Transendocardial	↔	↔	↓
CHART-1 [71]	2017	Autologous BM-MSC	IHF, LVEF ≤ 35%	Intramyocardial	↔	↔	N/A
ATHENA [72]	2017	Autologous ASC	ICM 20% ≤ LVEF ≤ 45%	Intramyocardial	↔	↔	N/A
MyStromalCell [73]	2017	Autologous ASC	ICM LVEF > 40%	Intramyocardial	N/A	N/A	N/A

↔↑↓ Respectively mean no change, increase and decrease. N/A means not measured

*BM* Bone marrow, *WJ* Wharton Jelly, *ICM* ischemic cardiomyopathy, *CABG* Coronary Artery Bypass Grafting, *LVEF* Left Ventricular. Ejection Fraction, *LVEDV* Left Ventricular End-Diastolic Volume, *ICM* Ischemic Cardiomyopathy, *STEMI* ST-elevation Myocardial Infarction

## Adipose tissue derived stem/stromal cells

ASCs were first discovered after their isolation from processed lipoaspirate by Zuk et al. in 2001 [19]. ASCs share many properties with bone-marrow MSC including their tri-lineage differentiation and the production of a variety of mediators. ASCs contribute directly to the homeostasis, tissue reparation and cell renewal in the adipose tissue. The International Fat Applied Society reached a consensus in 2013 regarding the minimum phenotypic criteria to characterize ASCs as CD39+, CD73+, CD44+, CD105+, CD90+, CD45−, CD31− plastic adherent stem/stromal cells. The expression of certain surface markers such as CD34 may change throughout cell division, meaning that different subpopulations of ASCs may exist in vivo [20]. They secrete factors that may inducing angiogenic and anti-apoptotic effects along with immunomodulatory properties. Therapeutic use of ASCs has thus far been promising in translational studies with encouraging data regarding safety and feasibility in clinical trials [21]. The interest in ASCs rests less on their differentiation capacity but rather on their ability to modulate their microenvironment by affecting injured cells through the release of a plethora of mediators.

## Direct mechanisms for cardiac repair: differentiation and fusion

The differentiation of MSCs/ASCs into cardiomyocytes, endothelial cells and vascular smooth muscle cells, the three main components of the cardiovascular system, have been previously been reported as achievable [22]. More specifically, in vivo differentiation of ASCs into cardiomyocytes has been documented since its first description [23]. After 3 weeks of treatment with 5-Azacytidine, cells showed spontaneous beating when observed under phase contrast microscope. Full phenotype characterisation showed positive staining for myosin heavy chain, α-actinin and troponin I. The differentiation was maintained up to 2 months. Cardiomyogenesis was also shown on ASCs spontaneously, with a pacemaker activity documented in electrophysiological studies on early ASC culture. Nevertheless, 5-Azacytidine induced cardiomyogenic differentiation remains controversial as such findings were not reproducible. Key cardiac marker expression such as cardiac troponin I and T and sarcomeric α-actinin as well as cardiac transcription factors GATA4 and Nkx2,5 were not detected [24].

Several studies have demonstrated in vivo engraftment and further differentiation into cardiomyocytes and endothelial cells after myocardial infarction (MI) [25, 26]. Yoon et al. reported engraftment and transdifferentiation of intramyocardially transplanted MSCs into cardiomyocytes [27]. Moreover, Valina et al. compared the intracoronary infusion of both BM-MSCs and ASCs on a porcine acute myocardial infarction (AMI) model. The group described similar efficacy of ASCs and BM-MSCs on cardiac function and angiogenesis but ASCs had better impact on LV remodelling [28]. This study also showed cell engraftment 4 weeks after transplantation with expression of endothelial cell markers CD31 and vWF in the engrafted cells. Fusion and mitochondrial transfer might also be another mechanism to cardiomyogenesis following ASCs transplantation [29]. One study showed in a murine AMI model, fusion of ASCs with cardiomyocytes with expression of connexin 43 and troponin I [30]. However, an important percentage of transplanted cells were lost through apoptosis or other mechanisms due to the harsh microenvironment in the infarcted area. Subsequently, it was also reported that a mild percentage of engraftment occurred without differentiation into cardiomyocytes, endothelial cells or smooth muscle cells [31]. Thus, it is unlikely that the transdifferentiation or cellular fusion of the low level of engrafted cells could account for the improved cardiac function after stem cell delivery.

## Autocrine effects

The autocrine activity of MSCs is induced by secreted factors acting on the stem cell itself. The majority of studies exploring the autocrine effects of MSCs are related to their differentiation capacity [32, 33]. Mediators in the conditioned media influenced differentiation capacity of MSCs or ASCs through an autocrine loop [34, 35]. For example, an autocrine signalling activity of VEGF-A was shown to influence osteogenic differentiation of human MSCs [36]. Another group demonstrated the importance of released FGF-2 and HGF on stemness of MSCs [37]. In addition, autocrine regulation has been described to influence immunomodulator mediators release. Stimulation of TLR3 on MSCs, which produced high levels of IL-6 and IL-8, upregulated TLR3 expression, hence inducing a positive feedback loop on IL-6 and IL-8 secretion [38]. Furthermore, autocrine effects can enhance survival or proliferation of stem cells in a hostile microenvironment. Lee et al. proved that PGE-2 secreted by human umbilical cord blood MSCs and ASCs plays a major role in maintenance of their self-renewal through EP2 receptor [39].

## Paracrine effects

It is now widely accepted that the main benefits of MSC therapy are derived from the effects of secreted factors acting on neighboring cells through a paracrine phenomenon. The diversity in the secreted factors constituting the secretome has been described and key factors have been identified such as VEGF, HGF, IGF-1, SDF1α, TGF-β and TSG-6 [40–42]. These mediators stimulate angiogenesis, inhibit apoptosis or modulate inflammatory pathways. Intramyocardial or intracoronary administration of stem cells are the routes for direct delivery that can permit paracrine effects on the injured myocardium. Bobi et al. used a porcine model of AMI and demonstrated increased gene expression of SDF-1α, GM-CSF and VEGF early on after intracoronary ASC injection. Enhancement of neovascularization is one of the most important therapeutic approaches needed to limit complications from post ischemic injury. Pro-angiogenic, antiapoptotic and anti-inflammatory effects have been described in this study [43]. Unfortunately, clinical studies used intracoronary administration of MSCs in acute myocardial infarction with contradictory findings, some showing improvements while others failed to report significant effects on either cardiac function or remodeling [44–46]. Intracoronary infusion has been preferred for the specificity of cell delivery to the target area. Caution has been raised regarding use of this route of delivery which might be associated with microvascular embolization leading to no-reflow phenomena. Nonetheless, recent clinical trials recognized safety of intracoronary injection of up to 50 millions of large size stem cells if injected a few days after myocardial infarction [47, 48].

Intramyocardial delivery has been associated with limited cell retention and engraftment. However, whether injected in the infarcted area or in the peri-infarcted zone, intramyocardial delivery of MSCs was able to ameliorate the infarct size. Perin et al. reported improved left ventricular ejection fraction (LVEF) and enhanced capillary density with transendocardial injection of allogenic MSCs in a canine AMI model [49]. However, Rigol et al. found that intracoronary infusion of ASCs improved neovascularization in porcine MI model compared to transendocardial demonstrating some conflicting results [26].

Yang et al. also concluded that the cardioprotective effect attributable to ASCs was mainly due to paracrine effects. They compared human ASCs vs. ASC-conditioned medium in a mice model of MI and observed a reduced infarct size, reduced cardiomyocyte apoptosis and improved cardiac function by both treatments. These results illustrated the sufficient impact of a cocktail of mediators injected in the peri-infarcted area [50].

Extracellular vesicles (EVs) are now recognized as important intercellular messengers involved in transmission of biological signals including proteins, lipids and RNAs. Exosomes are one subgroup of EVs originating from the fusion of multivesicular bodies and plasma membrane of stem cells and are retrieved in the secretome of MSCs of different origins. They are particularly enriched with mRNAs or micro RNAs (miRNAs) and have been investigated in acute kidney injury and ischemic disease such as stroke and myocardial ischemia reperfusion injury [51]. Some groups have experimented direct myocardial injection of MSC derived exosomes and observed reduced infarct size, preserved systolic/diastolic function and enhanced angiogenesis in a rat model of MI [52, 53]. Based on some reports, MSC-derived exosomes are accountable for the cardioprotective effects as their depletion from the conditioned media suppressed this protection [53]. This statement implies minor impact of cytokines and factors in the conditioned media of MSCs. It is presumed that exosomes are less prone to enzymatic degradation than the above-mentioned factors. The exact components of the cargo of exosomes that provide cardioprotection are yet to be discovered and characterized. It is fundamental to investigate efficacy difference between injecting conditioned media or MSCs, knowing that the advantage of cell injection is the responsiveness to the specific microenvironment whereas secretome can’t anticipate the pathological environment. It is even more complex knowing that some groups have demonstrated that not all exosomes are equivalent in their therapeutic impact [54, 55].

Endogenous cardiac progenitor cells in the myocardium are likely able to differentiate into cardiomyocytes, endothelial cells or smooth muscle cells to contribute to cardioprotection once activated. Given that their isolation and expansion is complex and still needs optimization for clinical application, stimulation and activation of endogenous progenitor cells by MSC secretome represents an advantage that can be exploited by cardiac cell therapy. Findings from in vitro and in vivo experiments have alluded to augmented differentiation and proliferation of cardiac progenitor cells through paracrine effects of MSCs [56, 57]. Release of SDF-1α and VEGF from transplanted MSCs and myocardial tissue was responsible for c-kit^+^ cell mobilization from the heart itself and from bone marrow to the infarcted region [57].

## Endocrine-like effects

An endocrine organ secretes hormones and factors that act at distance on other tissues. Factors need to circulate systemically to reach their specific receptors in order to intervene in their endocrine feedback loop. Aside from intracoronary and intramyocardial injections of MSCs discussed above, another route of delivery that has been tested in clinical trials is the intravenous injection, which is associated with ease of use and clinical translation. The pulmonary first pass effect has limited the number of cells reaching the infarcted myocardium when injected intravenously. Homing to the damaged tissue is then limited by entrapment in microvasculature when using this systemic administration [58]. Despite this seemingly disadvantageous side effect, Lee et al. have reported that entrapped MSCs in the lungs vasculature can induce therapeutic effects on the myocardium, reducing infarct size and improving cardiac function. These effects were attributed to various secreted factors but mainly TSG-6 [42]. This report remains a key finding hinting to an endocrine-like effect induced by secreted factors following cardiac cell therapy.

Intramuscular administration of stem cells is another remote delivery method that has been investigated for cardiac repair. In a rat dilated cardiomyopathy model, an intramuscular injection of human umbilical cord derived stem cells significantly elevated LVEF and left ventricular fraction shortening. Levels of circulating HGF, LIF, GM-CSF and VEGF were increased as well as the myocardial expression of HGF, IGF-1 and VEGF without stem cell homing to myocardium. Moreover, intramuscular injection into skeletal muscle did not induce any inflammation or ulceration in the tissue [59]. Intramuscularly administered MSC are largely trapped in the musculature without any detectable migration. A further advantage to the use of skeletal muscle as a repository for cell delivery is the ability to perform more than one injection without increased risk compared to more invasive methods. Remote delivery of MSCs into hind limb skeletal muscle has been found to improve ventricular function in a hamster heart failure model [60]. Elevated levels of HGF, LIF and G/M CSF as well as increased circulating c-kit cells, CD31+ cells and CD133+ cells were detected. Investigators considered the existence of a crosstalk between injected MSCs and endogenous bone marrow cells that would elicit increased activation of cardiac c-kit cells, involved in the cardiac repair. This crosstalk describes the dynamic and functionally relevant signalling pathways involved in the stem cell cardiac repair. Activated cardiac progenitor cells can further stimulate myocardial expression of paracrine factors.

Although subcutaneous MSC administration has been widely used in wound repair studies, its use in cardiac disease remains unexplored. Preda et al. hypothesized that a remote transplantation of ASCs transfected with heme oxygenase 1 could protect the heart from ischemia reperfusion injury. They reported that subcutaneously injected ASCs did not migrate systemically yet proliferated locally at the injection site. These genetically modified ASCs were able to improve cardiac functions post infarct despite their remote location from the site of injury [61]. Thus, Pentraxin 3 was identified as a possible mediator, acting in an endocrine-like manner to enact cardioprotection against ischemia reperfusion injury.

Human pharmacokinetic of biologic drugs is predictable when injected intravenously but this remains less clear regarding subcutaneous injections. Understanding the biodistribution and absorption of drugs delivered subcutaneously remains complex because of challenges to correlate preclinical findings in clinical setting due to the inherent difference in the subcutaneous tissue between humans and other species. Prediction of human pharmacokinetics following subcutaneous injection would rely on mechanistic studies rather than empirical scales. The implication of the lymphatic system in this route of delivery is also important. Blood capillaries are tight in their endothelial junctions, the transfer of macromolecules is then facilitated in the lymphatic capillaries as they have incomplete basal lamina which enables drainage of interstitial macromolecules without size restriction [62].

Exosomes as well as other trophic factors are the main actors of the immunoregulatory effect provided by MSCs. The main elucidated mechanisms in immunoregulation involve indoleamine 2,3-deoxygenase (IDO) or inducible nitric oxide synthase (iNOS), depending on the source of MSCs. IDO is an enzyme that catabolizes tryptophan and iNOS produces NO and both mediators can inhibit T cells [10]. Genetic knockdowns of these enzymes proved their implication in the immunosuppression, but the exact mechanisms are still unclear. Some direct interaction between MSCs and immune cells can also occur. Expressed factors on MSCs such as the co-stimulatory factors PDL1 and FASL can inhibit activated T cells by binding to CD80 [63].

The remote impact of MSCs on cardiac repair could be mediated by the activation of pericytes, microvascular mural cells. Pericytes are characterized by a strong regenerative ability, closely resembling MSC. Stem cell therapy has demonstrated that pericyte injection can reverse cardiac remodelling inhibiting fibrosis and inflammation while promoting angiogenesis [64]. One could hypothesize a crosslink between injected MSCs and resident pericytes that can potentiate the pro-healing impact of the cell transplantation. The involvement of pericytes could be their contribution to the pro-angiogenic effect of ASCs as the essential function of pericytes is to recruit and stabilize endothelial cells.

Finally, pericytes are also likely to participate in the regulation of the recruitment of immune cells following myocardial infarction, a role that has been described in other tissues [65]. Demonstrating efficiency of remote cell-based therapy would allow for non-invasive methods to treat ischemic heart disease. With the plethora of factors released following remote cell delivery, stem cells mobilized from niches such as bone marrow or activated endogenous cardiac progenitors can initiate and induce cardiac repair and/or regeneration following ischemic injury.

## Conclusion

**Fig. 2:**
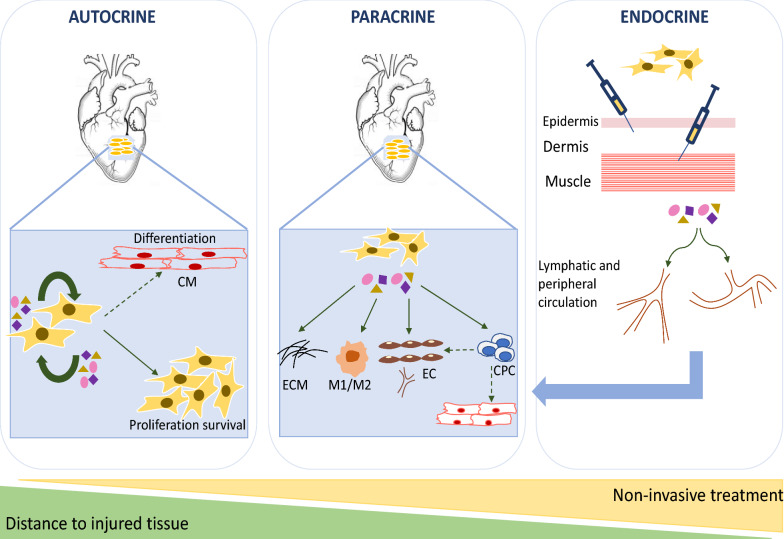
Repair pathways related to MSC-mediated therapeutic effects following ischemic injury. Autocrine pathways are involved in proliferation, survival and possible differentiation of MSCs. Paracrine pathways are elicited by secreted mediators that act in the vicinity of MSCs to promote angiogenesis, inhibit fibrosis and activate endogenous progenitor cells. Finally, endocrine-like pathways are induced when MSC (once remotely transplanted) are activated by distant injury and secreted trophic factors that circulate either in the vascular or lymphatic systems to induce pro-healing effects related to both autocrine and paracrine pathways

The therapeutic potential of MSCs/ASCs can be exerted through different mechanisms involving autocrine activity to enhance stemness, paracrine factors that improve local cardiac repair and endocrine-like effects to stimulate key stem-progenitor cells from their niches to further counter myocardial injury (Fig. 2). The most effective route of administration along with identification of the exact mechanisms of action of the trophic factors and exosomes remain to be uncovered in order to optimize clinical translation. Cardiac cell-based therapy, either via an invasive or remote manner, is indeed a promising adjuvant therapeutic strategy that can exploit advantageously the potency of the secretome of stem cells.

## Data Availability

Not applicable.

## Abbreviations

AMI: Acute myocardial infarction
ASC: Adipose derived stem cell
ESC: Embryonic stem cell
EVs: Extracellular vesicles
FGF-2: Fibroblast growth factor 2
G/M-CSF: Granulocyte macrophage colony stimulating factor
HGF: Hepatocyte growth factor
ICM: Ischemic cardiomyopathy
IHD: Ischemic heart disease
IGF-1: Insulin growth factor 1
iPSC: Induced pluripotent stem cell
LIF: Leukemia inhibitory factor
LVEF: left ventricular ejection fraction
LVEDV: Left ventricular end-diastolic volume
MI: Myocardial infarction
miRNA: Micro RNA
MSC: Mesenchymal stem cell
NO: Nitric oxide
SDF1α: Stromal cell derived factor 1a
TGF-β: Transforming growth factor B
TLR 3: Toll Like Receptor 3
VEGF: Vascular endothelial growth factor
